# Response of Estrogen Receptor-Positive Breast Cancer Tumorspheres to Antiestrogen Treatments

**DOI:** 10.1371/journal.pone.0018810

**Published:** 2011-04-14

**Authors:** Ada Ao, Brian J. Morrison, Heiman Wang, J. Alejandro López, Brent A. Reynolds, Jianrong Lu

**Affiliations:** 1 Department of Biochemistry and Molecular Biology, University of Florida College of Medicine, Gainesville, Florida, United States of America; 2 Queensland Institute of Medical Research, Royal Brisbane Hospital, Brisbane, Australia; 3 Griffith University, Nathan, Australia; 4 Department of Neurosurgery, McKnight Brain Institute, University of Florida, Gainesville, Florida, United States of America; Sanford-Burnham Medical Research Institute, United States of America

## Abstract

Estrogen signaling plays a critical role in the pathogenesis of breast cancer. Because the majority of breast carcinomas express the estrogen receptor ERα, endocrine therapy that impedes estrogen-ER signaling reduces breast cancer mortality and has become a mainstay of breast cancer treatment. However, patients remain at continued risk of relapse for many years after endocrine treatment. It has been proposed that cancer recurrence may be attributed to cancer stem cells (CSCs)/tumor-initiating cells (TICs). Previous studies in breast cancer have shown that such cells can be enriched and propagated *in vitro* by culturing the cells in suspension as mammospheres/tumorspheres. Here we established tumorspheres from ERα-positive human breast cancer cell line MCF7 and investigated their response to antiestrogens Tamoxifen and Fulvestrant. The tumorsphere cells express lower levels of ERα and are more tumorigenic in xenograft assays than the parental cells. Both 4-hydroxytamoxifen (4-OHT) and Fulvestrant attenuate tumorsphere cell proliferation, but only 4-OHT at high concentrations interferes with sphere formation. However, treated tumorsphere cells retain the self-renewal capacity. Upon withdrawal of antiestrogens, the treated cells resume tumorsphere formation and their tumorigenic potential remains undamaged. Depletion of ERα shows that ERα is dispensable for tumorsphere formation and xenograft tumor growth in mice. Surprisingly, ERα-depleted tumorspheres display heightened sensitivity to 4-OHT and their sphere-forming capacity is diminished after the drug is removed. These results imply that 4-OHT may inhibit cellular targets besides ERα that are essential for tumorsphere growth, and provide a potential strategy to sensitize tumorspheres to endocrine treatment.

## Introduction

The steroid hormone estrogen is central to the etiology of breast cancer. The biologic effects of estrogen are primarily mediated by estrogen receptors, namely ERα and ERβ [1]. In classic estrogen signaling, the binding of estrogen to ER causes receptor dimerization and binding to estrogen response elements (EREs) in promoter and/or enhancer regions of estrogen-responsive genes. Estrogen binding alters the three-dimensional structure of ER to facilitate recruitment of coactivator complexes, thereby activating the transcription of estrogen-inducible genes. The resultant transcriptional changes promote cell proliferation, survival, angiogenesis, and tumor metastasis.

ERα is a key transcriptional regulator in breast cancer and is responsible for many of the effects of estrogen on cancerous breast tissue. The majority of breast cancers are ERα-positive and depend on estrogen for growth [2]. Therefore, endocrine therapy that interferes with estrogen-mediated actions has been the strategy of choice for the treatment and prevention of ER-positive breast cancer. Clinically, inhibition of the estrogen signaling pathway is achieved mainly by targeting ER with selective ER modulators (SERMs) or the pure antiestrogen Fulvestrant, and blocking estrogen synthesis through aromatase inhibition [3], [4].

SERMs are synthetic molecules which bind to ER and modulate its transcriptional activity to block estrogen-stimulated breast cancer growth. Tamoxifen, the prototypical SERM, is the first-line therapy and a current standard adjuvant treatment extensively used for all stages of ER-positive breast cancer. 4-hydroxytamoxifen (4-OHT), the active metabolite of Tamoxifen, binds to ER in the same pocket as estrogen, but confers a conformation to the complex that is distinct from estrogen-bound ER. Consequently, binding of 4-OHT not only blocks association of coactivators but also recruits corepressors to prevent transcription of estrogen responsive genes [5]. Adjuvant therapy with 5 years of Tamoxifen reduces the disease recurrence rate by half and the annual breast cancer mortality rate by one-third, contributing significantly to the reduced mortality of estrogen-sensitive breast cancer [6]. Tamoxifen is also effective in prevention of breast cancer, decreasing its incidence by approximately 50% [7]. Fulvestrant/ICI182780 (ICI) has been approved as a second-line endocrine therapy for ER-positive breast cancer. ICI has a unique mode of action. It competitively binds to ER with high affinity and induces a conformational rearrangement that leads to accelerated degradation of ER protein [8]. ICI has shown equivalent clinical efficacy compared to Tamoxifen.

Endocrine therapy profoundly increases disease-free and overall survival in patients with ER-positive breast cancer. It has evolved to become the most effective and least toxic systemic therapy for this form of breast cancer. However, breast cancer recurrence after antiestrogen therapy has been a significant barrier for long-term positive outcome. Although Tamoxifen lowers the risk of recurrence for several years, late recurrences remain a major clinical challenge. Among women treated with a recommended 5-year Tamoxifen regimen, one-third of them would experience recurrent disease within 15 years [6].

There is increasing evidence that tumor persistence and recurrence may be attributed to cancer stem cells (CSCs) [9], [10], [11]. According to the CSC model, tumors are heterogeneous and many of them are organized as hierarchies in which a subpopulation of cancer cells, proposed as CSCs or tumor-initiating cells (TICs), possess stem cell-like properties. These cells can self renew as well as produce progenitors that rapidly proliferate and subsequently differentiate into diverse, more mature cell types that form the bulk of a tumor. TICs are intrinsically resistant to conventional chemo- and radiation therapies, and are able to regenerate the cellular components of the original tumor eradicated by such treatments, leading to recurrence. How to target and eliminate TICs is key to the design of more effective therapies.

It therefore becomes a critically important question how TICs from ER-positive breast cancer respond to endocrine therapy. Because *bona fide* markers for TICs are elusive, the exact identity of TICs remains contentious. Several complementary approaches have been used to enrich breast cancer TICs. The formation of mammospheres/tumorspheres in suspension culture is thought to be a hallmark of TICs [12], [13]. Serial passaging of spheres is an accepted measurement of self-renewal. Breast tumorspheres have been established from primary tumors and established cell lines. Breast cancer-derived sphere cells exhibit higher resistance to radiation and chemotherapy [14], [15], [16], and greater tumorigenic potential [17], [18]. However, it has not been reported whether these cells are responsive to anti-hormonal treatment.

The aim of this study was to characterize putative TICs from ER-positive breast cancer for their response to endocrine treatment in order to better understand and ultimately reduce tumor recurrence. In the present study, tumorspheres were established from ER-positive breast cancer cell line MCF7 and subjected to 4-OHT and ICI treatments. These treatments attenuated tumorsphere growth. However, after the treatments were stopped, the sphere formation frequency and tumorigenic potential of these cells remained unchanged. We further investigated the role of ERα in sphere growth and response to antiestrogens, and found that depletion of ERα unexpectedly sensitized tumorsphere cells to 4-OHT treatment.

## Results

### Tumorspheres derived from the MCF7 breast cancer cells exhibit increased tumor-initiating potential

**Figure 1:**
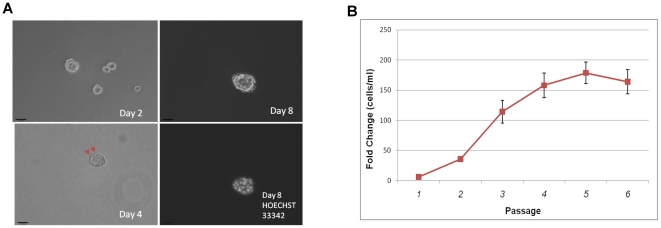
Establishment of tumorspheres from MCF7. **A**. Phase contrast images of tumorspheres derived from MCF7 cells 2, 4, and 8 days after initial seeding. Red arrows (bottom left) indicate microspikes, which are presumed to be microfilaments that spheroid cells use to sense nutrients in the environment. Hoechst nuclei staining (bottom right) shows a multicellular tumorsphere. Magnification at 100x. Scale bar  = 100 microns. **B**. Growth kinetics of MCF7S. Cells were seeded at 10,000 per ml and allowed to grow for seven days. The cells were then dissociated, counted, and passaged on the seventh day. This was repeated for 6 weeks. The experiment was performed twice with technical triplicates each time. Fold change is shown as final cell density/initial cell density.

The human breast cancer cell line MCF7 has been frequently used to isolate TICs, which grow as non-adherent tumorspheres [13], [14]. MCF7 cells were cultured under suspension conditions at 5,000 cells per ml in tumorsphere media [12], and they formed increasingly larger spheroids (Figure 1A). Eight days after initial plating, the cells formed tightly-packed, multicellular spheroids typically over 50 microns in diameter (Figure 1A).

Based on changes in cell proliferation kinetics (Figures 1B), the selection process appeared to be complete after the third passage (or the formation of tertiary spheroids). Cell proliferation rate became stabilized at approximately 200-fold change from passage 3 onward. These MCF7-derived tumorsphere cells were referred to as MCF7S.

**Figure 2:**
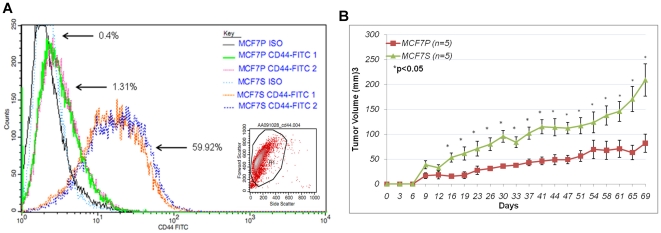
Increased tumorigenecity of MCF7S cells. **A**. Histogram of CD44-FITC and Iso-FITC staining for MCF7P and MCF7S. Duplicates are shown. Percentages of CD44 positive staining (from 30,000 cells) are indicated. Representative scatter plot and gating of FACS sorted cells is shown as inset. **B**. *In vivo* tumorigenic assay for MCF7 parental and MCF7S tumorsphere cells. Five mice were used for each group.

CD44 was previously described as a putative tumorigenic marker in breast cancer [19], and tumorsphere-forming cells were frequently enriched by isolating the CD44^high^/CD24^low^ cell population [16], [18], [20], [21], although the properties of the cell subgroup expressing CD44 and its specific role in tumor-initiation remain controversial. The status of CD44 in MCF7S and MCF7 parental cells was determined. Approximately 60% of MCF7S cells expressed the CD44 antigen while less than 2% of MCF7 parental cells expressed the marker (Figure 2A), suggesting that tumorsphere culture robustly enriched CD44-positive cells. Another marker, CD24, appeared to be slightly upregulated in MCF7S compared to the general population of MCF7 adherent cells (Figure S1). This discrepancy could be due to the heterogeneous nature of spheres, which contain differentiated cell types and variable proportions of TICs.

MCF7-derived tumorsphere cells were reported to be more tumorigenic than the parental cells [17]. The tumor forming potential of MCF7S cells was evaluated using *in vivo* tumorigenic assay. As shown in Figure 2B, tumors derived from MCF7S cells were significantly larger than those from parental MCF7 beginning 16 days post-injection, which continued to increase over time (e.g. Day 16 p = 0.011, Day 23 p = 0.041, Day 30 p = 0.005, Day 37 p = 0.005). MCF7S cells formed tumors more efficiently and had greater *in vivo* growth potential than the parental MCF7. These data suggest that the tumorsphere culture selects cells with tumorigenic potential.

### MCF7S cells retain ERα expression

Previous studies have argued that tumorsphere formation is associated with ERα-negative, basal cell types [22], [23]. More recent evidence suggests that a stem cell hierarchy exists during mammary stem cell development that supports the notion of a lineage-restricted, ERα-positive progenitor cell [24]. ERα-positive breast stem cells have been reported [25]. The parental MCF7 cells are positive for ERα, but it is unclear if ERα expression is altered during tumorsphere formation.

**Figure 3:**
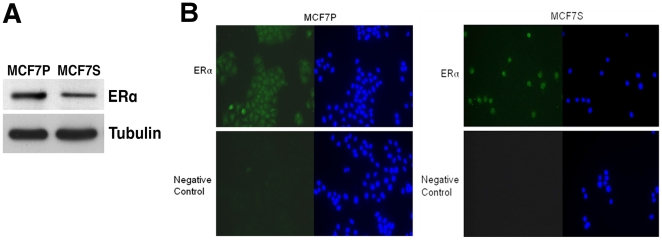
Estrogen receptor status in MCF7S. **A**. Immunoblotting of ERα protein in MCF7P and MCF7S cells. Tubulin served as loading control. **B**. Indirect immunofluorescence for ERα protein (green) in MCF7P and MCF7S. Cells were fixed by 3.7% formaldehyde. HOECHST 33342 (blue) was used to indicate nuclear region. A negative control was performed without primary anti-ERα antibody. Magnification at 40x.

With these conflicting viewpoints, ERα expression in MCF7S cells was examined and compared to parental MCF7 cells. Immunoblotting with anti-ERα antibodies detected the presence of ERα protein in MCF7S cells, but its level was modestly downregulated as compared to MCF7 monolayer cells (Figure 3A). Indirect immunofluorescence assay was used to determine ERα protein expression at the single cell level (Figure 3B). ERα was detected primarily in the nucleus of both MCF7 parental cells and MCF7S. Examination of hundreds of individual MCF7S cells showed that they were all positive for ERα (Figure 3B).

**Figure 4:**
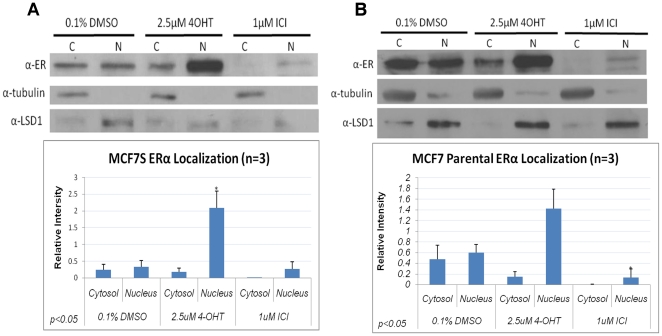
Effects of antiestrogens on ERα abundance and subcellular localization. Immunoblot of ERα protein in cytoplasmic and nuclear fractions from MCF7S (**A**) or MCF7 parental cells (**B**) treated for 48 hours with 4-OHT or ICI. Densitometry quantification of three independent experiments is shown below. Tubulin was used as a loading control for cytoplasmic (C) and LSD1 for nuclear (N) fraction. Statistically analysis was performed using paired Student's t-test.

Because of their ERα positivity, we investigated the effect of antiestrogens on the abundance and subcellular localization of ERα in MCF7S cells. Cell fractionation analysis was performed for both parental MCF7 and MCF7S cells after antiestrogen treatment for 48 hours. ERα protein was detected in MCF7S cells (Figure 4A). As expected, ICI downregulated overall ERα protein levels in both MCF7S and MCF7 parental cells. By contrast, 4-OHT strongly increased ERα protein in the nuclear fraction (Figure 4A and B), which is consistent with previous reports that 4-OHT stabilizes ERα in the nucleus [26], [27].

### Antiestrogens 4-OHT and ICI differentially attenuate tumorsphere formation and proliferation

**Figure 5:**
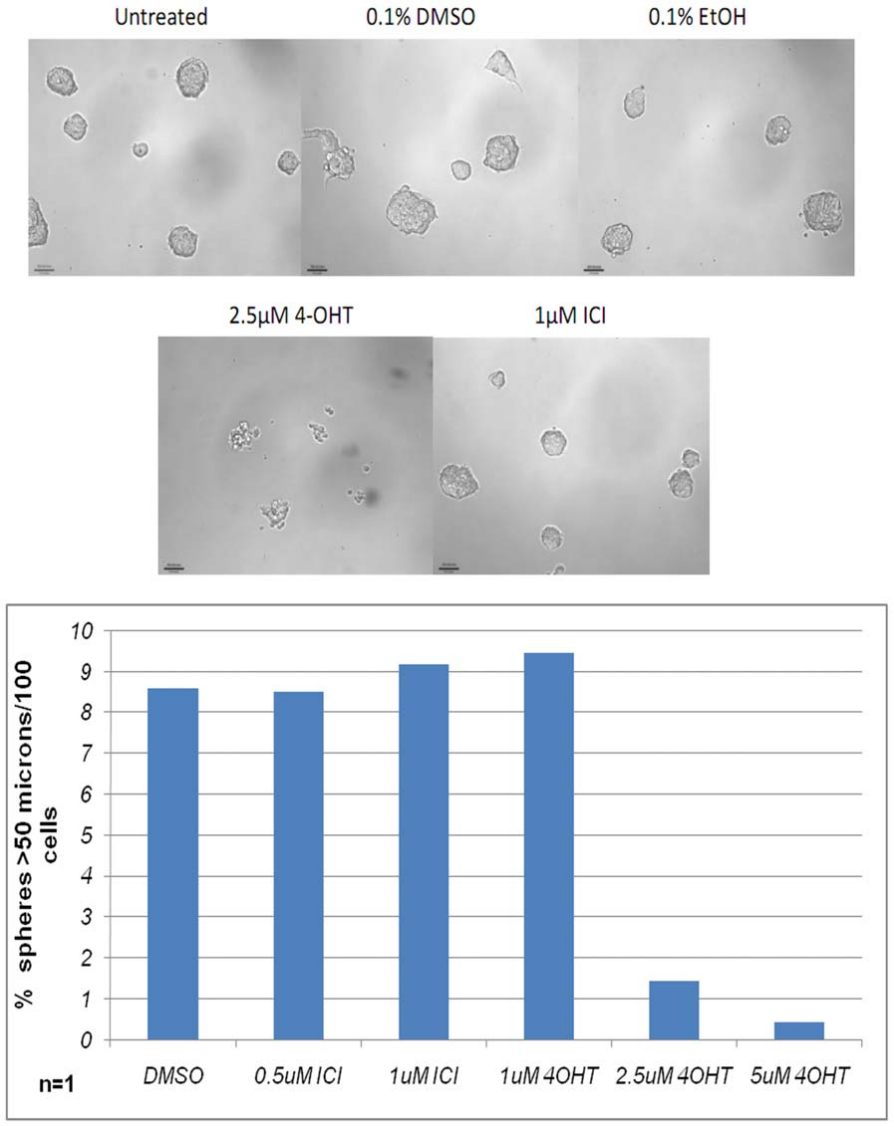
Effects of antiestrogens on tumorsphere formation. (Top) Phase-contrast images of MCF7S cells in the presence of antiestrogens or vehicle controls for 7 days. (Bottom) Quantification of MCF7S spheres with >50 micron diameter. Magnification at 100×. Scale bar  = 100 microns.

We next queried whether and how antiestrogen treatments might affect tumorsphere cells. MCF7S cells were treated with vehicle alone or singly with various concentrations of 4-OHT and ICI at the time of seeding. The cells were incubated for 6 days and tumorspheres were scored. As shown in Figure 5, treatment with 4-OHT at 2.5 µM or 5 µM remarkably disrupted tumorsphere formation and caused the cells to form disordered aggregates. However, cells exposed to vehicle controls, 1 µM 4-OHT, 0.5 and 1 µM ICI, still formed normal-looking spheroids. Therefore, the two classes of antiestrogens, 4-OHT and ICI, displayed different effects on sphere formation.

**Figure 6:**
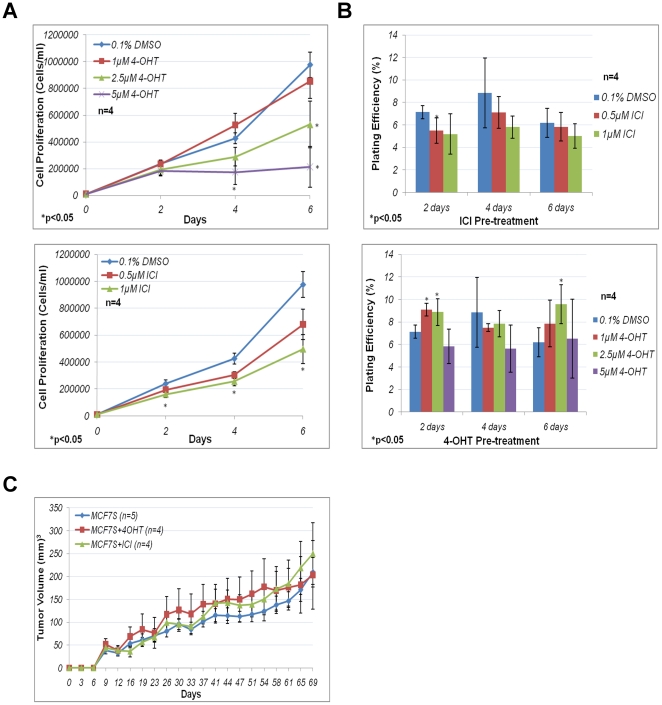
MCF7S antiestrogen response *in vitro*. **A**. Cell proliferation of MCF7S in the presence of antiestrogens. Cell proliferation of MCF7S treated with 4-OHT (top) or ICI (bottom) from four independent experiments. Error bars represent standard error of the mean (S.E.M.). **B**. Sphere formation of MCF7S after antiestrogen challenge with 4-OHT or ICI. (Top) Sphere formation frequency of 4-OHT-(top) or ICI-(bottom) treated MCF7S from four independent experiments. Plating efficiency was calculated as number of sphere >50 microns in diameter/total number of cells seeded ×100%. Error bars represent S.E.M. **C**. *In vivo* tumorigenic assay for MCF7S cells following antiestrogen challenge. MCF7S were either untreated, treated with 2.5 µM 4-OHT or 1 µM ICI for 4 days then recovered for 6 days in culture without antiestrogens, prior to injections. At least 4 mice were injected for each condition.

We further determined whether antiestrogens might influence MCF7S cell proliferation. The cells were treated with antiestrogens on the day of seeding. The cells were then counted 2 days, 4 days, or 6 days after seeding to determine the proliferation rate of these cells in the presence of antiestrogens. MCF7S responded to antiestrogens in a dose-dependent manner (Figure 6A). Proliferation of MCF7S cells were not significantly affected by 1 µM 4-OHT, while 2.5 µM 4-OHT significantly decreased cell proliferation by about 50% (p<0.05). MCF7S proliferation was essentially stopped by 5 µM 4-OHT. The effects of 1 µM ICI treatment were comparable to that of 2.5 µM 4-OHT for the inhibition of MCF7S proliferation (Figure 6A). MCF7S cells treated with 0.5 µM ICI showed a decrease in cell proliferation, although it was not significant when compared to 0.1% DMSO control (Figure 6A). Therefore, both antiestrogens at higher concentrations were capable of attenuating MCF7S proliferation.

### MCF7S sphere-forming and tumorigenic potential is unaffected after short term antiestrogen treatment

An interesting question was whether antiestrogen treatments might affect tumorsphere-forming potential. Therefore, MCF7S cells pre-treated with antiestrogens for various days were replated in media without drugs and sphere formation frequency was quantified. Approximately 5%–10% of bulk MCF7S cells were capable of sphere formation (Figure 6B). There was no statistically significant decrease in sphere formation efficiency following ICI treatment with the exception of 2 days 0.5 µM ICI treatment (p = 0.028). Sphere formation ceased in the presence of 4-OHT (2.5 µM or higher), but resumed when the drug was removed. There was a statistically significant increase in sphere forming frequency with 1 µM (p = 0.0004, 2 days) and 2.5 µM 4-OHT (p = 0.014, 2 days; p = 0.021, 6 days) treatments (Figure 6B). These results implied that sphere formation frequency of MCF7S cells remained essentially stable following ICI treatment, and 4-OHT might select for cells with mildly increased sphere-forming potential.

Tumorsphere formation correlates with enrichment of tumor-initiating cells and may serve as an indicator of tumor-forming potential [15], [18]. *In vivo* tumorigenic assay was performed in immune deficient mice to evaluate tumorigenic potential of MCF7S cells following antiestrogen challenge. MCF7S cells were pre-treated for 4 days, recovered in fresh media for 6 days, and subjected to xenograft transplantation. The resulting tumors showed no significant differences in tumor size when compared to untreated control (Figure 6C), implying that antiestrogen treatments did not alter the tumorigenic potential of MCF7S cells.

### MCF7S cells retain self-renewal capacity in long-term antiestrogen treatment

According to the CSC/TIC hypothesis, self-renewing TICs only grow and divide asymmetrically to maintain homeostasis, and the growth of the bulk population is dependent on non-tumorigenic cells. If MCF7S does indeed contain a subpopulation of TICs, then it is possible to examine this property through long-term serial passage. This method, which has been used in neural stem cells characterization [28], can characterize the effects of antiestrogens on long-term self-renewal of tumorigenic cells.

This was achieved by serially passaging viable cells for multiple passages. The cells were treated either with vehicle alone or antiestrogens at the time of seeding, and incubated for 7 days as constituting a single passage. Viable cells were counted at the end of each passage. The fold change in cell number for each passage was used to calculate potential expansion of the population if all the cells, instead of a fraction, were maintained in culture. The long term growth kinetics was compared between different antiestrogen treatments.

**Figure 7:**
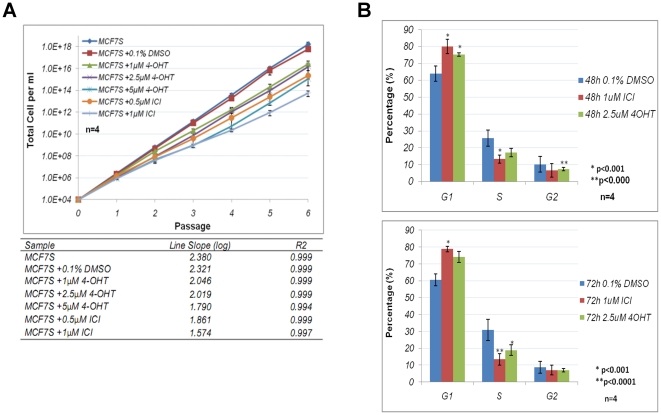
Effects of antiestrogens on long-term growth and cell cycle of MCF7S. **A**. Long-term expansion of MCF7S in the presence of antiestrogens. The lines are expressed on a semilog graph (top) and slope of each line was calculated as log of averaged expansion (bottom). Data were derived from four independent experiments. Error bars: S.E.M. **B**. Propidium iodide (PI) cell cycle analysis of 48 (top) and 72 (bottom) hours antiestrogen treated MCF7S. Data were derived from four independent experiments and analyzed using ModFit LT software. Error bars: S.E.M.

Control treatment (0.1% DMSO) did not influence long term cell expansion of MCF7S (Figure 7A). There was a decrease in long term expansion for 1 µM and 2.5 µM 4-OHT treated MCF7S, and 5 µM 4-OHT and ICI treated cells showed the most dramatic decrease in long term expansion (Figure 7A). It is evident that all antiestrogen treatments reduced MCF7S long term growth. The results indicated that decrease in long-term cell expansion did not correlate with sphere formation frequency.

Cell cycle analysis with propidium iodide staining further confirmed the changes in growth rate. Cells treated with 4-OHT or ICI had a significant increase of G1 phase cells and a significantreduction of S phase cell population when compared to vehicle-treated control cells (Figure 7B). These results suggested that antiestrogen treatments attenuated cell cycle progression, which might contribute to the decrease in long-term expansion.

### ERα is dispensable for sphere formation or tumorigenic potential *in vivo*

ERα is the primary target of antiestrogens. The evidence that tumorsphere formation persisted in the presence of ICI suggested that ERα might not be essential for sphere formation, although it could influence cell proliferation in bulk tumorsphere culture. Therefore, it was necessary to examine ERα's role in the context of potential TICs.

**Figure 8:**
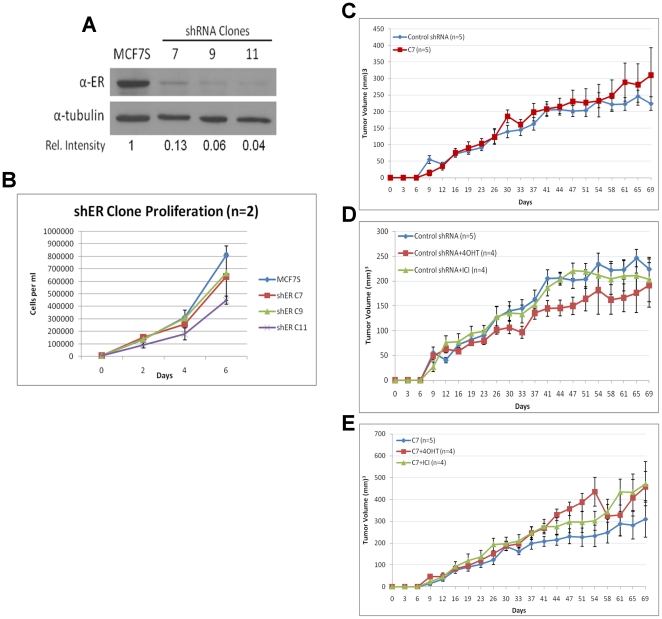
ERα is disposable in MCF7S. **A**. Immunoblot analysis of ERα in three shERα MCF7S clones. Relative densitometry intensity is shown. **B**. Cell proliferation of three shERα clones compared to bulk MCF7S culture. Error bars represent standard deviation from two experiments. **C**. *In vivo* tumorigenic assay comparing shRNA control and shERα knockdown MCF7S. Five mice were injected for each condition. **D** and **E**. *In vivo* tumorigenic assay comparing antiestrogen treated (4 days) shRNA control or shERα MCF7S cells. At least 4 mice were injected for each condition.

ERα was specifically depleted using shRNA via retroviral transduction. Three stable knockdown (shER) clones with efficient depletion of ERα were identified (Figure 8A). Compared to bulk MCF7S cells, clone 11 exhibited much lower proliferation rate, but clones 7 and 9 did not show significant reduction in growth (Figure 8B). All three shER clones were capable of forming spheres at sizes and frequencies similar to those of control MCF7S cells (not shown), confirming that ERα was not required for tumorsphere formation or maintenance.

We further determined whether ERα was required for the tumorigenic potential of MCF7S cells. *In vivo* tumorigenic assay was performed to compare retroviral-mediated ERα knockdown cells to control shRNA-transduced cells. There was no statistically significant difference in tumor volume between control shRNA transduced MCF7S and shER MCF7S cells (Figure 8C). In addition, following antiestrogen pre-treatment, both control shRNA and shER transduced MCF7S cells showed no statistical differences in tumor growth (Figure 8D and E), which is analogous to data from bulk MCF7S (Figure 6C). In conclusion, these observations suggest that ERα is dispensable for sphere formation and tumorigenicity of MCF7S.

### Depletion of ERα sensitizes MCF7S cells to 4-OHT treatment

**Figure 9:**
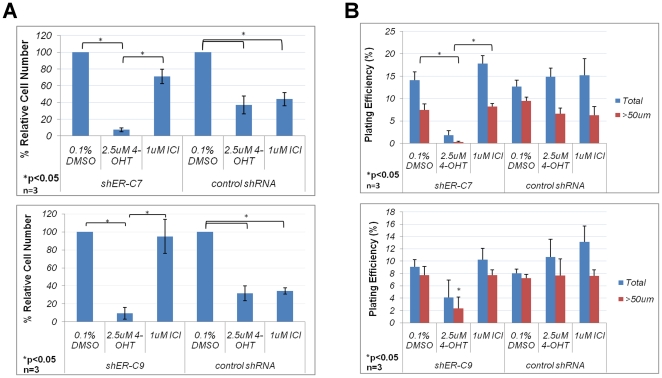
Antiestrogen response of ERα knockdown MCF7S cells. **A**. *In vitro* antiestrogen response of ERα knockdown in MCF7S cells. % Relative Cell Number represents viable cell number in antiestrogen-treated samples relative to vehicle-treated control. Data for two individual clones were generated from three independent experiments. Error bars represent S.E.M. **B**. Sphere formation frequency of antiestrogen treated ERα knockdown MCF7S cells. Three independent experiments were performed for two individual clones. Error bars: S.E.M.

The indication that ERα is not required for sphere formation raised questions about its role in MCF7S' antiestrogen response. The ERα knockdown clones were further characterized for proliferation and sphere formation frequency under antiestrogen treatment. All three clones exhibited similar responses. Unlike the control shRNA MCF7S cells, the shERα clones demonstrated diminished response to ICI, as the compound had no significant effect on cell number (Figure 9A). This indicates that ERα has been sufficiently depleted in the shERα cells to nullify ICI's effects and that ICI's activity depends on the presence of ERα. Unexpectedly, the shERα cells exhibited a much heightened sensitivity to 4-OHT treatment (Figure 9A).

The sphere formation capacity of the shERα clones was evaluated as described for Figure 6b. The ERα knockdown clones pretreated with vehicle or ICI for 6 days showed similar sphere formation frequency to that of control shRNA MCF7S (Figure 9B). However, a dramatic reduction in sphere formation potential was observed in shERα cells treated with 4-OHT (Figure 9B). Because sphere formation is a measurement of TIC self-renewal capacity, this finding implies that 4-OHT treatment may be able to shrink the pool of TICs in MCF7S cells depleted of ERα.

## Discussion

Adjuvant endocrine therapy of ER-positive breast tumors is an important advance in breast cancer treatment. These hormonal interventions reduce the disease recurrence rate, however, many patients treated with the therapies still suffer relapse years later. A better understanding of the mechanisms underlying tumor recurrence should improve therapeutic efficacy and reduce mortality from ER-positive breast cancer. The concept of cancer stem cells responsible for tumor recurrence has increasingly gained prominence in breast cancer research. This model suggests that only a minority population of cells in tumors, termed CSCs/TICs, possess extensive self-renewal potential and are able to sustain tumor growth, metastasis, and recurrence [9], [10], [11]. To eliminate residual breast cancer disease after endocrine therapy may require effective targeting of this cell population. The CSC/TIC theory has profound implications for our understanding of tumor recurrence and for the design of novel treatments. However, how the TICs from ER-positive breast tumors react to antiestrogen treatment were unclear.

The TICs are recognized by their capacity to grow as tumorspheres *in vitro* and initiate tumor formation *in vivo* [12], [13], [19]. In this study, tumorspheres derived from the ERα-positive MCF7 breast cancer cell line were used as a model to characterize their response to antiestrogen treatment. Tumorsphere cells demonstrated increased tumorigenicity when transplanted into immunocompromised mice than the bulk parental cells. The sphere cells retained ERα expression (albeit at reduced levels when compared to parental cells) and were responsive to antiestrogens. Acute antiestrogen treatment with 4-OHT or ICI decreased their proliferation. However, the treated cells retained the same capability of sphere formation *in vitro* and tumorigenicity *in vivo*. After withdrawal of the drugs, tumorsphere growth resumed. The comeback tumorsphere cells remained sensitive to antiestrogens as repeated, longer-term antiestrogen treatment inhibited cell expansion. There was no indication of strong enrichment of antiestrogen resistant cells. This is consistent with previous findings that antiestrogen-resistant clones are rare and require remarkably prolonged selective pressure *in vitro* to isolate [29]. It should be pointed out that the concentrations of Tamoxifen in breast tumors from patients under endocrine therapy are mostly at 300–400 ng/g [30], or approximately 1 µM, which is slightly lower than that was used in this study. The requirement of higher levels of Tamoxifen could be attributed to the source of cells. Ideally, tumorspheres directly derived from ER-positive clinical tumor samples will be examined for their sensitivity to antiestrogen treatment. Consistent with our *in vitro* observation, recent clinical studies demonstrate the positive impact of extended endocrine therapy on suppressing late recurrences in ER-positive breast cancer [31], [32].

Our study suggested that ERα was not essential for tumorsphere formation or tumor growth, implying that TICs do not rely on ERα for self-renewal (even though they may express detectable levels of ERα). Surprisingly, ERα-depleted tumorspheres are hypersensitive to 4-OHT treatment. It is likely that 4-OHT has additional target(s) (other than ERα) in these cells that also contribute to tumorsphere formation. Such target(s) may be preferentially co-expressed with ERα, but their identity remains unknown. Nevertheless, this observation that depletion of ERα sensitizes tumorspheres to 4-OHT may be explored to target TICs. On the other hand, this result does not indicate that Tamoxifen is more effective on ER-negative tumors. It is widely appreciated that ER-positive breast cancers have fundamentally distinct clinicopathologic and genetic characteristics when compared to ER-negative cancers [6], [33], [34], [35]. The benefit of Tamoxifen is so far limited to the group of women with ER-positive tumors. Simple depletion of ERα does not change other properties of ER-positive tumor cells and make them equivalent to ER-negative tumors.

Tumorsphere formation only enriches TICs, and the enrichment of TICs may be variable. In a given sphere, only 5–10% of the population possesses the self-renewal capacity to form new spheres when plated (Figure 6B). If these cells represent TICs, the majority of the bulk sphere cells are more differentiated cells [12]. The heterogeneity in MCF7S seems to be rigidly maintained, as the proportion of self-renewing cells remains almost constant over multiple passages and after antiestrogen treatment (Figure 6B). This homeostasis may suggest that non-TIC cells may form a niche to support the maintenance of TICs. The exact ERα status in different cell subpopulations in spheres remains to be determined. TICs may express sufficient levels of ERα and are direct targets of antiestrogens. It is also possible that TICs only express minimal amount of ERα and are hence intrinsically insensitive to antiestrogens. In this case, antiestrogens may target non-TIC cells in spheres and disrupt the niche for TICs.

## Materials and Methods

### Antiestrogens, antibodies and shRNA constructs

4-Hydroxytamoxifen and ICI 182780 were purchased from Sigma. Anti-ERα antibody for immunoblotting was purchased from Santa Cruz (clone HC-20, SC543), and clone MC-20, SC542 was used for immunofluorescence. CD44-FITC (clone G44-26, 555478, BD Pharmingen) and Iso-FITC (MG2b01, Molecular Probes) were kind gifts from Drs. Johannes W. Vieweg and Sergei Kusmartsev.

Oligos for shRNA constructs were designed using psm2 designer at RNAi Central (http://katahdin.cshl.edu/siRNA/RNAi.cgi?type=shRNA). The shRNA oligos were cloned into the retroviral pLMP vector (OpenBiosystems).

### Tumorsphere generation and formation assay

Tumorsphere media was composed of 50∶50 DMEM/F12 media supplemented with 20 ng/ml EGF, 10 ng/ml bFGF, 5 µg per ml heparin, 1% penicillin-streptomycin solution, 1% L-glutamine, and 1X B-27 supplement [12]. MCF7 parental cells from ATCC were seeded at 5,000 living cells per ml in defined tumorsphere media onto Poly-HEMA coated dishes [12]. The cells were passaged once a week to select for tumorsphere culture cells. The culture was maintained without additional growth factor supplementation or culture media change over the course of a week from initial plating until the next passage for secondary spheroids.

MCF7 tumorsphere single cell suspension was diluted to 5,000 cells per ml and plated onto 96-well plate (approximate 500 cells per well). The cells were incubated for 6 days and phase contrast pictures were taken. Sphere size and number were manually measured and counted from the images using ImageJ software. Statistical analysis was performed using Student's t-test.

### Expression of CD44 and CD24 by flow cytometry

Approximated 1×10^6^ cells were trypsinized to obtain single cell suspension. The cells were washed twice in cold PBS + 1% BSA, and subsequently incubated at 4°C with either 1∶25 diluted CD44-FITC antibody, CD24-FITC antibody, or isotype-FITC control antibody in PBS + 1% BSA for 45 minutes in the dark. After incubation, the cells were washed twice in cold PBS + 1% BSA and resuspended in 400 µl cold PBS + 1% BSA for flow cytometry analysis within 1 hour.

### *In vivo* tumorigenesis

All mouse work was conducted according to relevant national and international guidelines. The Queensland Institute of Medical Research Animal Ethics Committee (QIMR-AEC) approved this project under protocol number P1159. 7–9 week old NOD.Cg-*Rag1^tm1Mom^Il2rg^tm1Wjl^*/SzJ mice (The Jackson Laboratory, Bar Harbor, Maine) were used for the subcutaneous (s.c.) injections of breast cancer cells. One day before transplantation, the mice received 60-day release 17β-estradiol pellets (Innovative Research of America, Sarasota, Florida) placed s.c. in the interscapular region. Mice received 2×10^6^ cells mixed 1∶1 in PBS and matrigel (BD). Mice were monitored twice a week for overall health. Tumor diameters were measured with a digital caliper and tumor volume in mm^3^ was calculated using the formula: Volume  =  width^2^ × length ×0.5. The results were compared using unpaired t-test.

## Supporting Information

Figure S1 **CD24 expression in MCF7P and MCF7S cells.** Histogram of CD24-FITC and Iso-FITC staining for MCF7P and MCF7S. Duplicates are shown. 10,000 cells were examined for each staining.
(TIF)
