# Supplementary figures and images for: Response of Estrogen Receptor-Positive Breast Cancer Tumorspheres to Antiestrogen Treatments
Source: PLoS One. 2011 Apr 14;6(4):e18810. doi: 10.1371/journal.pone.0018810 (PMC3077404; doi:10.1371/journal.pone.0018810)

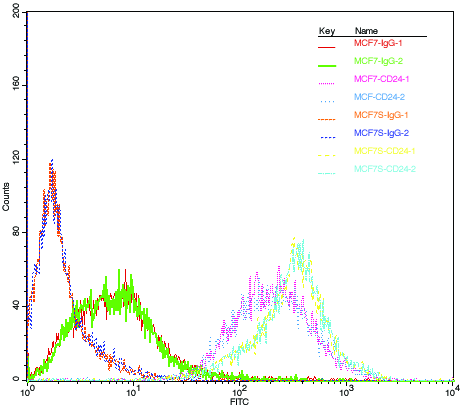

Supplement: Figure S1 — CD24 expression in MCF7P and MCF7S cells. Histogram of CD24-FITC and Iso-FITC staining for MCF7P and MCF7S. Duplicates are shown. 10,000 cells were examined for each staining. (TIF) [file pone.0018810.s001.tif]
